# The Luxury of Giving

**Published:** 1891-01-03

**Authors:** 


### THE LUXURY OF GIVING.

"I like Christmas," said a six-year-old model of Miss Dorothy Tennant to the sympathetic woman artist. "Santa Claus puts things in one's stocking then, I found a halfpenny in mine last Christmas. But," he added, as a regretful afterthought, "I had put it there myself the night before."

There was something very forlorn in the poor little fellow's attempt to get up the Christmas feelings he had heard about, those sensations of joyous expectation and pleased surprise that come as a matter of course to happier children; and it is a little pitiful to think how many people, young and old, are more or less in the baby Whitechapeller's position. Except, indeed, that they cannot get up even an imitation of the "comfort and joy" of Christmastide by gifts to themselves of either great or small amount. They know who put the halfpenny there; they cannot pretend that it is a token of love from either saint or sinner. And, after all, it is love that we desire, and the gifts are precious only in so far as they reveal it. Love, indeed, cannot be bought in the shops, but there are ways of earning it. Just fancy, for example, what a spring of affection would have burst from Miss Tennant's little model if some of the earthly representatives of Santa Claus had put in his stocking, say, a whole bright silver threepenny bit! And the banking account of the said representative would not have been really so very much diminished. One of the most touching and most terrible revelations of the loneliness and gloom of so many of our brethren's lives comes in their deep and permanent appreciation of little acts of kindness. There is deep truth, beautiful yet sad, in Wordsworth's lines:—

> "I've heard of hearts unkind, kind deeds
> With coldness still returning;
> Alas! the gratitude of men
> Hath oftener left me mourning."

With all our cheap cynical talk about the ingratitude of the poor, we cannot but be conscious of their real thankfulness for genuine kindly help, and if it sometimes saddens us it is chiefly because our conscience speaks, and says how easily that gratitude might have been won long ago. Now with the New Year comes the time for a fresh start. Some of us, indeed, must set our minds to thinking of the economies we must effect this year, but others are calculating on the luxuries they may indulge in. We commend to their notice one of which perhaps they have not thought—the luxury of giving. Not the cheap luxury of throwing a penny to a beggar as we pass; that is the lazy charity that hurts and does not help. It is not merely a luxury, it is an extravagance. All good economists like to know where their money goes; to have something to show for it. In spending on others as much as in spending on oneself, thought and inquiry are needed. Of course it is not always possible for individuals to investigate thoroughly the course pursued by every shilling they give. Once—only once—we heard a lady say that she knew no poor people, and though few are in her exceptional position, a good many are so placed that direct gifts of money, clothing, or food, are comparatively little required by any of the poor they know. It is the task—often more difficult—of obtaining suitable work for the parents and education, cheap or free, for the children that falls upon many people in their private charities, and the time and trouble necessary for this busy folks cannot give. So they remain, almost with purse in hand, unable to indulge in the luxury of giving, because they do not personally know suitable recipients for their gifts. But surely such hesitation is needless. Let those who give nothing on the plea that "there are so many calls upon them that they cannot respond to all," decide in what class of the suffering community—surely it is large enough!—they are most interested. Is it orphans or the aged; the sick, the lame, or the blind; is it those whom poverty has led into sin; or those who yet cling, desperate yet brave, to honesty and virtue; is it those whose intellect wants only guidance or use, or those whom Providence has sent into the world with faculties impaired? Choose the object of your charity; you need do no more. There are plenty of institutions, tried and warranted, that care for all of these. It is not easy to diminish appreciably the mass of human misery, but we may say with some satisfaction that there is no form that misery takes which is not dealt with by some of our charitable institutions. Choose then whom you will help; the way of assistance is simple and clear; and you will taste one of the luxuries that never pall—the luxury of giving.

